# Histological Study of Suprabranchial Chamber Membranes in Anabantoidei and Clariidae Fishes

**DOI:** 10.3390/ani11041158

**Published:** 2021-04-17

**Authors:** Dobrochna Adamek-Urbańska, Ewelina Błażewicz, Magdalena Sobień, Robert Kasprzak, Maciej Kamaszewski

**Affiliations:** Department of Ichthyology and Biotechnology in Aquaculture, Institute of Animal Science, Warsaw University of Life Sciences, Ciszewskiego 8, 02-786 Warsaw, Poland; blazewicz.ewelina96@gmail.com (E.B.); sobienmag@gmail.com (M.S.); robert_kasprzak@sggw.edu.pl (R.K.); maciej_kamaszewski@sggw.edu.pl (M.K.)

**Keywords:** air-breathing fishes, fish histology, ARO, respiratory epithelium, gills

## Abstract

**Simple Summary:**

Air-breathing fish constitute a broad evolutionary group of fish, which are generally characterized by distinctive phenotypical plasticity. These fishes usually inhabit waters where oxygen deficiency occurs periodically, which is why they have developed a variety of accessory respiratory organs (AROs) that may be used in an obligatory or a facultative manner. Knowledge of the structure of these organs is important for both the breeding and the conservation of these fish species. The aim of this study was to conduct a comparative histological analysis of two types of AROs found in the Anabantoidei suborder and the Clariidae family, both of which are freshwater fish taxa of high ecological and commercial importance.

**Abstract:**

Accessory respiratory organs (AROs) are a group of anatomical structures found in fish, which support the gills and skin in the process of oxygen uptake. AROs are found in many fish taxa and differ significantly, but in the suborder Anabantoidei, which has a labyrinth organ (LO), and the family Clariidae, which has a dendritic organ (DO), these structures are found in the suprabranchial cavity (SBC). In this study, the SBC walls, AROs, and gills were studied in anabantoid (*Betta splendens*, *Ctenopoma acutirostre*, *Helostoma temminckii*) and clariid (*Clarias angolensis*, *Clarias batrachus*) fishes. The histological structure of the investigated organs was partially similar, especially in relation to their connective tissue core; however, there were noticeable differences in the epithelial layer. There were no significant species-specific differences in the structure of the AROs within the two taxa, but the SBC walls had diversified structures, depending on the observed location. The observed differences between species suggest that the remarkable physiological and morphological plasticity of the five investigated species can be associated with structural variety within their AROs. Furthermore, based on the observed histology of the SBC walls, it is reasonable to conclude that this structure participates in the process of gas exchange, not only in clariid fish but also in anabantoids.

## 1. Introduction

Fish, like other animals, breathe oxygen, and its availability is crucial for their survival. An aquatic environment offers a much lower oxygen content compared to atmospheric air, which is why fish evolved special anatomical structures that enable efficient oxygen uptake from the water. Evolutionally, the ability to breathe atmospheric air was first achieved among vertebrates by fish, which occurred around 400 million years ago [1]. This ability arose independently in different evolutionary lines of fish, and the manner of air-breathing which developed in Sarcopterygians was inherited by the tetrapods. On the other hand, in the Actinopterygii clade, air-breathing mechanisms continue to be supported by dissolved oxygen uptake from the water. While, in some species, this ability has regressed with time, in others, it either remains active to the present day or was reacquired after the preceding regression [2,3]. In the latter group of fish, oxygen uptake takes place not only through the gills, a special form of respiratory organ equivalent to the lungs of terrestrial vertebrates, but also through accessory respiratory organs (AROs), which are frequently found in fishes living in poorly oxygenated waters throughout the world [4]. Some of the most well-known representatives of air-breathing fish are species belonging to the suborder Anabantoidei (order: Anabantiformes) and the family Clariidae (order: Siluriformes).

The Anabantoidei, commonly called labyrinth fish, consist of either three or five families, depending on the methodological approach of the taxonomists. Anabantidae, Helostomidae, and Osphronemidae are the main families, as indicated by genetic-based research [5,6,7]; however, in classical descriptions, the subfamilies Luciocephalinae and Belontiinae were distinguished as separate families from the Ospronemidae [5,8,9,10]. The natural habitats of labyrinth fishes are found in the tropical waters of Africa and South Asia, in various aquatic ecosystems characterized by constant or periodic low oxygen content [11]. All of these species are characterized by high phenotypic and morphological plasticity, which, during the life of the fish, allow it to adapt to changing environmental conditions [12]. This developmental plasticity is mainly driven by environmental conditions, such as the content of dissolved oxygen in water, and not by genetic determinants [13,14].

The Anabantoidei develop a labyrinth organ (LO) late during their growth, sometimes even after the attainment of sexual maturity [15], until which the gills remain the leading respiratory organ. The LO is located in the suprabranchial cavity (SBC), which is fully separate from the buccal cavity (unlike in its sister suborder Channoidei [7,16]), and is a plate-based organ covered by a heavily vascularized respiratory epithelium. The entire structure is flexible and may be supported by loose connective tissue and cartilage and/or bone. In some fish species, muscle tissue also occurs within the organ. The location and morphology of the LO also depend on the overall size of the body, as well as on the structure and size of the skull [1]. The origin of the LO has been investigated before, and the first hypothesis was that this organ evolved from a gill-derived structure [17]. It was later verified by Hughes and Munshi (1973) [18] that pillar cells in the AROs are not of the same type as in the gills, which probably indicates morphological differences between these organs.

A structure similar to the LO, the dendritic organ (DO), is found in members of the Clariidae family, commonly known as air-breathing catfish, which belong to the Otocephala clade and are significantly distinct from the Anabantoidei in terms of phylogeny (clade Euteleostei) [19,20,21]. Therefore, this is an indication that these catfish evolved their own type of ARO following the mechanism of parallel evolution, while the secondary loss or reduction of this organ in some clariid genera was described as an ecophenotypic variation [22]. The Clariidae use the DO to absorb atmospheric oxygen during the seasonal drying of African and Asian tropical swamps and rivers [20,21]. These fish are only facultative air-breathers, in contrast to Anabantoidei; however, as suggested by Damsgaard et al. (2020) [8], air-breathing in Clariidae may be associated with a variable metabolic rate. With circadian changes in its metabolism, the African sharptooth catfish (*Clarias gariepinus*, Burchell, 1822) breathes more intensively during the night, and this increases simultaneously with the rise in its metabolic rate. The efficiency of this organ is associated with the presence of highly vascularized structures occurring not only in the DO itself, but also in the wall of the suprabranchial cavity chamber [23].

The DO forms near the second and fourth gill arches and has a structure which bears similarity to a tree or shrub [24]. This organ is located at the posterior of the gill arches, with the smaller fragment located on the second gill arch and the larger on the fourth. The general structure of the dendritic organ resembles that of the LO in the Anabantoidei—there is a strongly vascularized, respiratory epithelium, with pillar-like cells and mucocytes, all supported by connective tissue [25]. The DO is connected to the gills through the same cartilage from which the branches of gill arches deviate [20]. The presence of a cartilaginous core ensures the relatively high durability of the DO during drought, in contrast to the gills, which collapse in the absence of water, due to being non-rigid [26].

A thin, respiratory epithelium completely covers both the LO and the DO, where it rests on a basal plate, and is generally similar to the gill epithelium, which is beneficial for the diffusion of gases to and from the capillaries [26,27]. The epithelial layer also includes mucocytes responsible for: (a) providing optimal conditions for gas exchange, (b) protecting the delicate respiratory epithelium from all kinds of impurities, and (c) preventing particles suspended in water or air from interacting with the gills or the superficial cavity, possibly causing micro-injuries [26].

The respiratory mechanism of the discussed fish species is not limited only to the gills, LOs, or DOs, because additional parts of these organs have also specialized to support this process. The walls of the gill and suprabranchial cavities are constructed similarly due to their common embryological origin during craniofacial development. Furthermore, the suprabranchial chamber is not only the location of the respiratory organs, but, in some fish species, this structure also actively participates in the processes of hearing [28], digestion [29], or breathing [1,8,23,30,31,32].

Hitherto, there is a lack of histological comparison between the AROs of Asian and African Clariidae and representatives of the Anabantoidei. The need to obtain knowledge about the anatomy and physiology of these fishes appears to be crucial for two major reasons. Firstly, some of these fish species are of great local (and sometimes global) economic importance, either in aquaculture or in the ornamental fish trade. Secondly, in an era of globalization and the prevalent lack of clear and transparent international provisions regarding animal transportation and introduction, the invasiveness of some of these air-breathing species will increase significantly, especially more so during a climate catastrophe. It is therefore crucial to expand our basic knowledge about the anatomy and physiology of such fish species, so that, in the future, it can be used effectively to improve means of protection, breeding, and reproduction of aquatic wildlife. Therefore, the aim of this study was to compare the histological structure of the LO, DO, and SBC wall and expand the current knowledge about these air-breathing fishes. This comparison could allow for a better understanding of the structure of the AROs and may possibly allow us to learn more about the mechanisms of evolutionary solution that determined the ecological plasticity of fish and their adaptation capacity to the ever-changing environmental conditions.

## 2. Materials and Methods

This study was carried out in accordance with the guidelines provided by the 2nd Warsaw Local Ethics Committee for Animal Experimentation, residing at the Warsaw University of Life Sciences.

Post-juvenile individuals of five fish species were used in the study: the Siamese fighting fish (*Betta splendens*, Regan, 1910), the leopard bushfish (*Ctenopoma acutirostre*, Pellegrin, 1899), the kissing gourami (*Helostoma temminckii*, Cuvier, 1829), the Angolian walking catfish (*Clarias angolensis*, Steindachner, 1866), and the Philippine catfish (*Clarias batrachus*, Linnaeus, 1758). For each species, the material was taken from five randomly purchased individuals (Table 1).

Fish were slaughtered by decapitation with prior stunning. Whole fish were fixed in 4% neutral buffered formalin (NBF) and decalcified in Leica Decalcifier II (Leica Biosystems, Nussloch, Germany). Samples were subjected to a standard paraffin procedure [33] and were cut longitudinally and sagittally with the Leica RM2265 (Leica Biosystems, Nussloch, Germany) microtome at 5 µm thickness. Standard procedure was conducted to stain the slides with hematoxylin and eosin (HE), and, based on initial results, additional stainings were carried out. The collagens and intracellular matrix components were stained with Mallory trichromate (Supplementary Data 1). To visualize the mucosa cells and blood vessels, a modification of the standard AB/PAS (pH 2.5) procedure was performed (Supplementary Data 1). The slides were analyzed with a Nikon Eclipse NI-E microscope with a Nikon DS-Fi3 camera and NIS Elements AR software (Nikon, Tokyo, Japan).

Morphometric analysis of the epithelial height was performed in all investigated fishes. For each of the four studied locations (the cranial, dorsal, and caudal parts of the SBC wall, as well as the LO/DO), a total of 50 measurements per group were conducted. Statistical analyses were performed with Statistica 13.3 software. Differences between parts of the SBC were analyzed for significance with a t-test (*p* < 0.05) and displayed as means with their standard deviation (±SD).

## 3. Results

### 3.1. Gills and Gill Cavity

**Figure 1:**
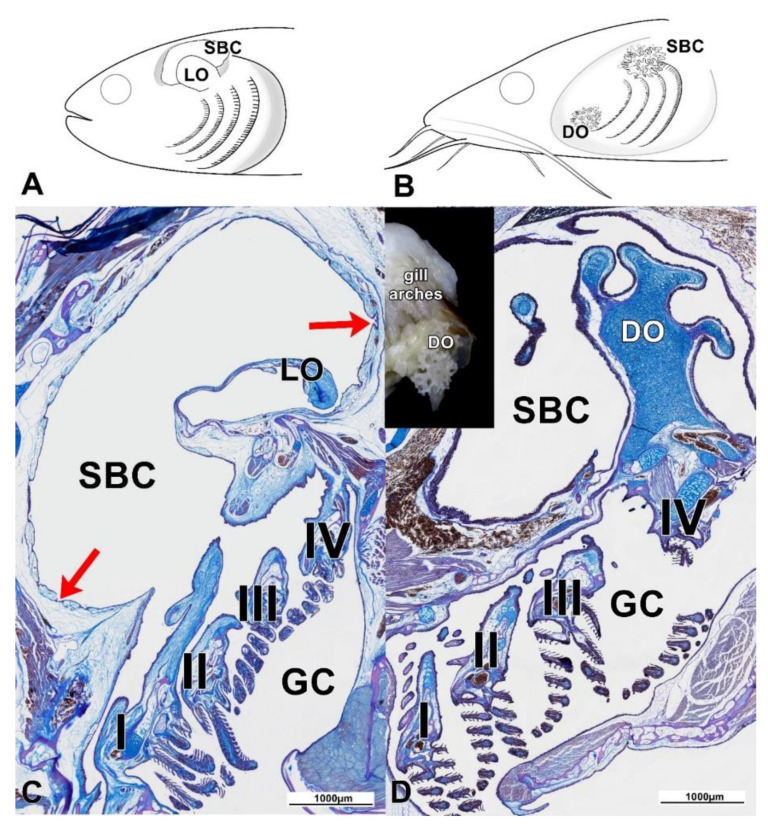
(**A**,**B**) General anatomy of the gill and suprabranchial cavities of Anabantoidei (**A**) and Clariidae (**B**). (**C**) In the Anabantoidei species, the suprabranchial cavity was lined with a special type of epithelium, with multiple cavities (red arrows). The labyrinth plate was supported by bone and thick connective tissue, with collagen fibers strongly stained with aniline blue. (**D**) The dendritic organ of the Clariidae was supported by fibrous cartilage and a thinner connective tissue layer (compared to the Anabantoidei specimens). LO—labyrinth organ; DO—dendritic organ; SBC—suprabranchial cavity; GC—gill cavity; Roman numerals label the gill arches; modified AB/PAS, scale 1000 µm.

The gills of all the investigated individuals were located in gill chambers (Figure 1A,B), with the cores of the gill arches (bony in Claridae, cartilaginous–bony in Anabantoidei) covered by a thin layer of loose connective tissue, along with primary and secondary lamellae (Figure 1C,D).

**Figure 2:**
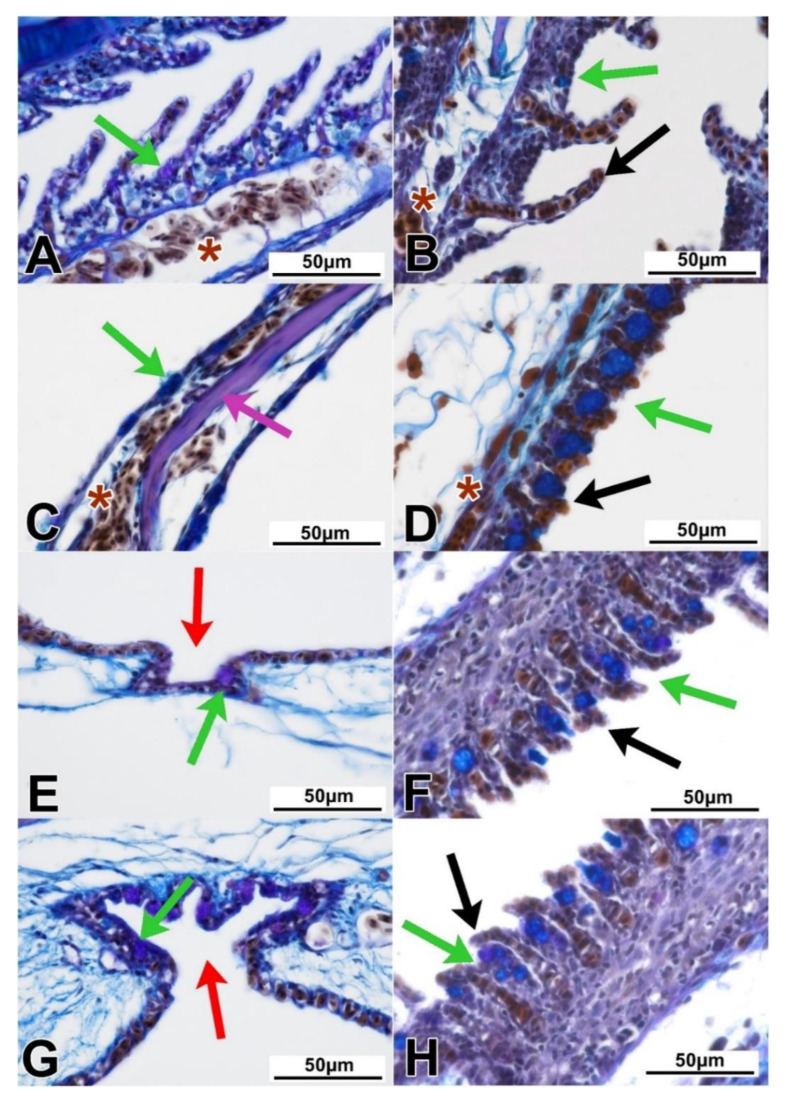
Histological details of the gills and accessory respiratory organ parts in the Anabantoidei (**A**,**C**,**E**,**G**) and the Clariidae (**B**,**D**,**F**,**H**) fish species. (**A**) The primary and secondary gill lamellae in the Anabantoidei were covered by a simple epithelium with mucous cells and blood vessels. (**B**) A similar epithelium was found in the gills of the Clariidae. (**C**) In the labyrinth organ, the bony core was not far beneath the epithelium. (**D**) The cartilaginous core of the dendritic organ was cushioned by a thick layer of connective tissue. (**E**) Caveolae found in the cranial part of the SBC wall of the Anabantoidei. (**F**) Cranial part of the SBC wall in the Clariidae. (**G**) Caveolae found in the caudal part of the SBC wall of the Anabantoidei. (**H**) Caudal part of the SBC wall in the Clariidae. Asterisks—blood vessels; green arrows—mucous cells; red arrows—caveolae; black arrows—capillaries/capillary vessels; magenta arrow—bony core of labyrinth; modified AB/PAS; scale—50 µm.

In all investigated individuals, different types of gill rakes were presented, with the most compound structures (acting as a filtration organ) found in the kissing gurami fish. The filtratory rakes were structured similarly to gills, with their cartilaginous core, connective tissue, and single-layered epithelium clearly visible. The lanceolate-shaped, branched gill rakes were observed as having a bony core and acidic mucosal cells. In the secondary lamellae, the highly vascularized, single-layered epithelium contained mucosal cells with acidic, neutral, and mixed mucus (Table 2; Figure 2A,B).

### 3.2. Labyrinth Organ (LO) and Suprabranchial Cavity of Anabantoidei Species

**Figure 3:**
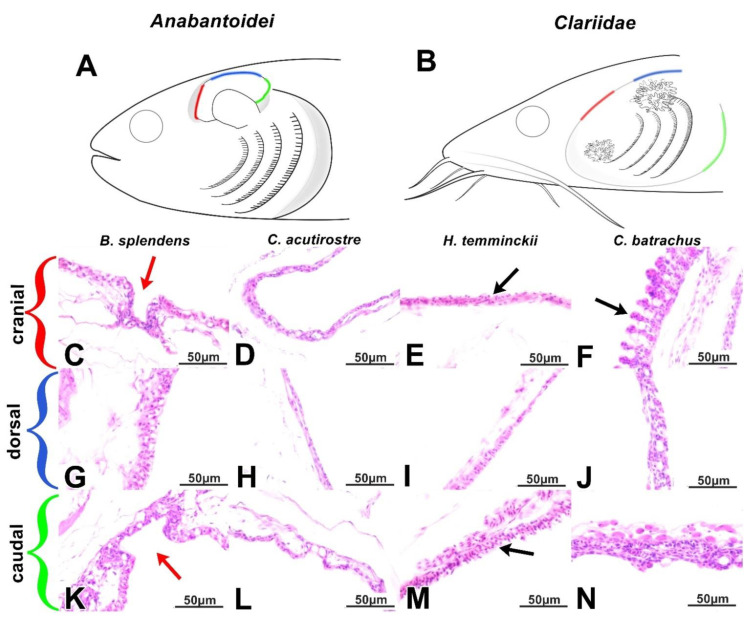
(**A**,**B**) The main differences in the SBC walls between the Anabantoidei (**A**) and the Clariidae (**B**) were distinguished between the cranial (red), dorsal (blue), and caudal (green) parts of this anatomical structure. (**C**–**F**) The described structure was less developed in the cranial parts of the Anabantoidei (**C**–**E**) when compared to that of the Clariidae (**F**). (**G**–**J**) The dorsal parts of the SBC walls. (**K**–**N**) The caudal parts of the SBC walls. Red arrows–caveolae; black arrows—extensions; HE stain; scale—50 µm.

The LOs of all the studied fish species had a mixed, cartilaginous–bony core. Each of its elements was surrounded by loose connective tissue, covered by a single-layered epithelium (Figure 1C). In this organ, as in the case of gills, mucous cells were identified. Both the LOs and gills were characterized by a distinct network of blood vessels (Figure 2C). The LOs of *C. acutirostre* and *B. splendens* were similarly structured, but the cartilaginous core of these two species was characterized by a more compact structure than in *H. temminckii*, indicating partial ossification (pink arrow in Figure 2C). In the epithelium of the LO and the suprabranchial cavity, both acidic (blue) and neutral (magenta) mucous cells were identified (Figure 2C). In the suprabranchial wall of *B. splendens*, cavity-like structures were observed, with multiple mucosa cells found at their bottom, and without any visible blood vessels surrounding these structures (black arrows for caveolae, green for mucosa cells, Figure 2E,G). The complexity level of these caveolae was species-specific (the most complex were found in *B. splendens*) and varied depending on their localization. In the anterior and posterior parts of the suprabranchial wall, the caveolae were the most complex, while in the dorsal part they were the least complex (Figure 3C–E,G–I,K–M). The mean epithelial height values in the cranial, dorsal, and caudal parts of the SBC wall were significantly different (*p* < 0.05) in all investigated anabantid species (Table 3).

Furthermore, in the suprabranchial cavity walls of all three Anabantoidei species, small, extension-like structures (similar to gill lamellae) were observed, as distinguished from the caveolae. The structure and length of these protrusions/extensions varied in terms of location, but a pronounced network of blood vessels was always found in their immediate proximity (Figure 3C–E,G–I,K–M).

### 3.3. Dendritic Organ (DO) and Suprabranchial Cavity of Clariidae Species

The DOs of both Clariidae species were divided into two parts; the smaller one was located in the gill cavity, while the larger and more widespread part was located within the suprabranchial cavity and located caudo-dorsally in relation to the gills (Figure 1B,D). The structure of the DOs was similar between species, regardless of the studied location. Elastic cartilage formed the core, covered by a thin layer of loose connective tissue and lined with a strongly vascularized, respiratory epithelium (Figure 1D and Figure 2D). In some of the sections of the DOs, a different kind of cartilage was noticed, with irregularly shaped cells and centrally arranged nuclei. In addition, the amount of basic substance in-between the chondritic territories was noticeably less than that of typical hyaline cartilage. Both cartilage types were surrounded by loose connective tissue as described above (Figure 2D). In the epithelium covering the DO fans, there were mostly acidic mucous cells, stained dark-blue with Alcian blue (Figure 2D). The walls of the gill cavity were structured differently than in the Anabantoidei species. Moreover, morphological differences were also observed depending on the location of the observed epithelia. In the anterior parts of the wall, instead of cavities as in Anabantoidei, a thin respiratory epithelium was observed, with numerous tongue-like capillaries and mucous cells (Figure 2E–H). In the centripetal and caudal parts, mucous cells were present, and the epithelium was similar to that of the oral cavity or esophagus. Folded extensions, similar to the shorter ones observed in the Anabantoidei in the anterior and caudal parts of the suprabranchial cavity membrane, were observed in the caudal area in Clariidae (Figure 3K–N). The mean epithelial height values in the cranial, dorsal, and caudal parts of the SBC wall were all significantly different (*p* < 0.05) in both investigated clariid species (Table 3).

## 4. Discussion

The ichthyofauna of oxygen-poor ecosystems developed several evolutionary solutions in order to improve the efficiency of gas exchange [1]. For instance, numerous modifications of hemoglobin and other blood components facilitated their adaptation to different oxygen levels, both in air and water [34,35]. Other physiological adjustments included various metabolic strategies which allowed them to withstand endogenous ammonia toxicity during emersion [36]. Meanwhile, histoanatomical solutions are associated with the presence of the respiratory epithelium not only in the gills but also in the gill-derived AROs [25,37], or even in the posterior intestine [38]. This epithelium is usually single-layered, with abundant mucous cells, and is, overall, a delicate structure that is fixed to its basal lamina and underlying connective tissue (rich in collagen), both of which provide proper mechanical support and necessary elasticity of the whole membrane [39]. On a larger scale, the gills are arches located in the gill cavity with a cartilaginous or bony core, with numerous extensions in the form of filaments and lamellae to maximize the gas exchange surface [40]. Likewise, some parts of the AROs may also have a core made of cartilage, bone, or an intermediate type of skeletal tissue, but the exact tissue type depends on the species. For instance, in fish from the Clariidae family, the presence of a hyaline cartilage core, as was the case in both species in this study, is normal [41]. Furthermore, fishes also developed muscular elements in their AROs, which set these structures in motion (fully or at least to some extent) and therefore impact the air ventilation of their respiratory chambers [42]. However, few researchers pay specific attention to the structures of the gill and suprabranchial cavities, both of which may serve a prominent respiratory function, as hypothesized for fishes belonging to both Clariidae [43] and Anabantoidei [31]. The extensions presented on the DO’s surface for both Clariidae species are thin-walled structures which, when exposed to air, assume shapes described as finger-like [26], balloon-like [31], tongue-like [20], or globular [26]. Due to the inflow of blood into these structures through the capillaries, their shape changes, increasing the surface area accessible for gas exchange. Meanwhile, when the gills are able to maintain sufficient blood oxygenation on their own, the blood flow through the DO capillaries is reduced, restricting the ability of red blood cells to reach the tips of these finger-like lamellae, which is why these structures enter a “resting state”, lying flat on the surface of the DO [26]. Such a situation was observed in this study, in the dorsal and caudal sections of the suprabranchial membrane of the two Clariidae fish species. This resting mechanism provides a barrier between atmospheric air in the suprabranchial chamber and blood in the respiratory lamellae in the DO [26]. Considering the fact that in Clariidae, the anterior part of the gill cavity, along with the entire surface of the suprabranchial cavity wall, contains numerous structures similar to the gills and identical to those of the DO; it can be assumed that the suprabranchial cavity’s epithelium itself may also participate in gas exchange.

Presumably, the outgrowths found in the wall of the suprabranchial cavity of the studied Anabantoidei species could also have a respiratory function, similarly as the structures found in Clariidae fish. The highest complexity among these structures was found in the cephalic and caudal segments of the cavity of the Siamese fighting fish and kissing gourami. The cells presented in these protuberances were not only erythrocytes but also pillar-like cells similar to those co-forming the respiratory epithelium of the gills [44,45]. Therefore, these numerous extensions in the suprabranchial cavity wall correspond to the ”islets” described by Munshi (1968) [46] as locally occurring structures resembling lamella II of the gills. However, this similarity is justified further, as analogous structures have been demonstrated in the respiratory islets of the *Clarias* and *Heteropneustes* genera [8]. Therefore, it may be assumed that this membrane, similar to that of Clariidae, probably also participates to some extent in gas exchange.

Mucous cells were present in large numbers in the both of the studied ARO types because they are key producers of the surface surfactants responsible for enhancing gas exchange. Surfactants, as in alveoli in tetrapod lungs, reduce surface tension, which promotes more efficient gas exchange [47]. They also have a protective effect by coating the respiratory epithelium with a protective layer, reducing the adhesion of foreign particles [48], and acting as an antimicrobial and antiradical cover [49]. Depending on the chemical composition of the secretions, mucins can stain with both Alcian blue and Schiff’s reagent. In the present study, mucous cells presented in the suprabranchial cavity epithelium in all investigated individuals were characterized by a differentiated response in AB/PAS staining, although cells with acidic or mixed secretions were predominant. The amount of acidic mucins in the mucus correlates with the degree of hydration of the respiratory epithelium, due to its high water-binding capacity. A histochemical analysis of the DOs of African catfish [25] demonstrated mucous cells producing acidic and mixed mucins, similar to the examined *C. batrachus* and *C. angolensis*. Meanwhile, neutral mucus has a lower viscosity compared to acidic mucus and provides excellent protection against physical damage [50], but has a lower preventive potential against parasites and microorganisms [51].

Mucous cells were also present in the described cavities/caveolae in the Anabantoidei, with few blood vessels in close proximity. A similar structure was described in the study of Olson et al. (1994) [24], which identified two types of structures of a respiratory and non-respiratory nature in the buccopharynx wall in *Channa punctatus*. The respiratory structures consisted of squamous epithelium with erythrocytes and mucous cells “protruding” above the epithelial line, while the non-respiratory part was characterized as a “papillated surface”. All sections of the suprabranchial cavity of the Anabantoidei fish were organized in a similar manner—as alternating or continuously occurring epithelial tissue fragments with or without protuberances, and with or without mucous cells. Blood vessels were present underneath the individual respiratory sections, as in *C. punctatus* [32], indicating the convergent evolution of similar adaptations in different organs, with differences in the LOs being a feature likely correlated with respiratory performance and habitat occupation, rather than taxonomy [15].

## 5. Conclusions

In conclusion, to date, morphological research has not indicated that the suprabranchial cavity wall in investigated labyrinth fishes directly participates in air respiration, a feature which was discovered in Clariidae [25]. Acknowledging the fact that its structure and structural organization partly resembles both the LO and the DO in Clariidae, with the current state of knowledge, it seems highly probable that such an active respiratory role of the suprabranchial epithelium occurs also in the Anabantoidei. In the future, it appears plausible to carry out further analyses which take into account the detailed structure of the suprabranchial cavity wall and its potential physiological importance for these fish.

## Figures and Tables

**Table 1:** Morphometrical parameters of body weight and length of investigated species.

Species	Body Weight (g)		Length (cm)	
	Average	SD	Average	SD
*Betta splendens*	0.99	0.23	4.32	0.20
*Ctenopoma acutirostre*	0.91	0.06	3.64	0.09
*Helostoma temminckii*	2.55	0.36	5.80	0.27
*Clarias angolensis*	8.31	1.09	11.47	0.80
*Clarias batrachus*	3.04	0.73	8.04	0.69

**Table 2:** Types of mucous cells in different parts of the walls of the respiratory organs in the investigated fish species.

Species	Gills	Gill Chamber	Labyrinth Organ	Dendritic Organ
*B. splendens*	acidic, mixed	mixed	acidic, mixed	N/A
*C. acutirostre*	acidic, mixed	acidic	acidic, mixed	N/A
*H. temminckii*	acidic	mixed	acidic	N/A
*C. angolensis*	acidic	acidic, mixed	N/A	acidic
*C. batrachus*	strongly acidic	acidic, mixed	N/A	acidic, neutral (few)

**Table 3:** Epithelial height in the cranial, dorsal, and caudal parts of the suprabranchial cavity and LO or DO in investigated species.

	Epithelial Height (µm)							
Species	Cranial		Dorsal		Caudal		LO/DO	
	Average	SD	Average	SD	Average	SD	Average	SD
*B. splendens*	11.13 *	5.32	14.04 *	5.25	10.85 *	2.64	6.19	1.61
*C. acutirostre*	10.26 *	3.08	4.02 *	1.36	15.28 *	4.07	6.65	1.81
*H. temminckii*	17.19 *	6.38	7.46 *	1.34	13.86 *	5.68	7.80	1.87
*C. angolensis*	31.97 *	8.65	22.06 *	5.17	39.96 *	11.87	28.36	5.88
*C. batrachus*	23.66 *	3.40	21.00 *	4.25	33.43 *	8.03	22.72	4.78

* statistically significant differences between the parts of each particular species (*p* < 0.05).

## Data Availability

Data which was not presented in this manuscript or supplementary material is available on request from the corresponding author.

## Supplementary Materials

The following are available online at https://www.mdpi.com/article/10.3390/ani11041158/s1, Supplementary file 1.
