# Supplementary material for: Histological Study of Suprabranchial Chamber Membranes in Anabantoidei and Clariidae Fishes
Source: Animals (Basel). 2021 Apr 17;11(4):1158. doi: 10.3390/ani11041158 (PMC8073562; doi:10.3390/ani11041158)
Supplement: Supplementary file 1 [file animals-11-01158-s001.zip › animals-1166342-SI.pdf]

Supplementary data 1:

Hematoxylin & eosin staining protocol:

1. Deparaffinized and rehydrated slides were incubated for 10 minutes in Ehrlich's hematoxylin[i].
2. Differentiation was conducted in acidic alcohol (0.5% HCl in 70% EtOH).
3. Slides were washed in tap water (pH 8.4) for 5 minutes.
4. Counterstaining in aqueous 0.5% eosin solution for 5 minutes.
5. Differentiation in 70% ethanol for 10 seconds.
6. Dehydration, clearing (with xylene) and mounting (with DPX).

AB/PAS staining protocol:

1. Deparaffinized and rehydrated slides were washed in deionized water.
2. Staining in 1% (pH 2.5) Alcian blue for 10 minutes.
3. Slides were washed in tap water for 5 minutes and then rinsed in deionized water.
4. Incubation in 0.5% periodic acid for 5 minutes.
5. Washing in deionized water was repeated 3 times, for 1 minute each.
6. Staining in Schiff's solution (Sigma Aldrich) for 15 minutes in 4°C.
7. Slides were washed in tap water for 5 minutes.
8. Counterstaining with Harris' hematoxylin[ii] for 2 minutes.
9. Differentiation in acidic alcohol (0.5% HCl in 70% EtOH).
10. Dehydration, clearing (with xylene) and mounting (with DPX).

Mallory's trichrome staining protocol:

1. Deparaffinized and rehydrated slides were washed in deionized water.
2. Staining in aqueous 0.5% acidic fuchsin solution for 7 minutes.
3. Staining in Mallory's solution[iii] for 20 minutes.
4. Differentiation in 96% alcohol.
5. Quick dehydration, clearing (with xylene) and mounting (with DPX).

Modified AB/PAS combined with Mallory's staining protocol:

1. Staining in 1% (pH 2.5) Alcian blue for 10 minutes.
2. Slides were washed in tap water for 5 minutes and then rinsed in deionized water.
3. Incubation in 0.5% periodic acid for 5 minutes.
4. Washing in deionized water was repeated 3 times, for 1 minute each.
5. Staining in Schiff's solution for 15 minutes in 4°C.
6. Slides were washed in tap water for 5 minutes.
7. Counterstaining with iron hematoxylin[iv] for 2 minutes.
8. Differentiation in acidic alcohol (0.5% HCl in 70% EtOH).

9. Incubation in Mallory's solution for 10 minutes.
10. Differentiation in 96% alcohol.
11. Dehydration, clearing (with xylene) and mounting (with DPX).

---

[i] 1g hematoxylin was dissolved in 100ml of 99% ethanol. 100 ml of 3% ammonium alum was added and 100ml of glycerin. To acidify, 10 ml of glacial acetic acid was added. For oxidation, sodium iodate was added (0.01g).

[ii] Two solutions were mixed: 1% alcoholic hematoxylin with warmed saturated ammonium alum. For oxidation, mercuric oxide was added (0.25g). After cooling, 0.2 ml of glacial acetic acid was added.

[iii] 0.5g aniline blue was mixed with 2g of orange G and 1g of phosphotungustic acid in 100ml of deionized water.

[iv] 2% alcoholic hematoxylin was mixed with equal volume of 4% iron chloride before staining.
